# Editorial: Qualitative research on therapist-client interaction in psychotherapy, volume II

**DOI:** 10.3389/fpsyg.2023.1285978

**Published:** 2023-09-15

**Authors:** Yijin Wu

**Affiliations:** School of Translation Studies/Center for Medical Humanities in the Developing World, Qufu Normal University, Rizhao, China

**Keywords:** qualitative research, therapist, client, psychotherapy, interaction

The exchange between therapists and clients play a crucial role in psychotherapy, serving as its foundation during therapy sessions. Recently, there has been a growing emphasis on qualitative analysis of therapist-client interaction within the field (Peräkylä, 2011, 2019; Wu, 2019, 2021). This shift in focus recognizes that the nature of the therapeutic relationship, as expressed through verbal and nonverbal communication, profoundly influences therapy outcomes. Qualitative analysis allows for a closer examination of various factors, such as dialogue patterns, levels of empathy and rapport, and therapeutic techniques employed. Through this approach, researchers can capture the subtle nuances and inherent complexities that shape the therapeutic alliance. Additionally, qualitative analysis permits the exploration of how different therapeutic modalities and interventions impact the dynamic between therapists and clients. By examining the intricate interplay between language, emotions, and nonverbal cues, researchers can unveil the mechanisms that contribute to meaningful change and improvements in clients' wellbeing. This Research Topic encompasses qualitative analysis of therapist-client interaction across various types of psychotherapy.

In a study conducted by Voutilainen et al., they used Conversation Analysis to explore how empathy and challenge in therapist-client interactions influence psychophysiological responses. Through the analysis of data collected from psychodynamic therapy sessions, the authors discovered that both empathy and challenge influenced the emotional arousal of participants. Therapists' empathy was found to reduce clients' emotional arousal while increasing their own throughout the session. Conversely, when therapists presented challenges during interventions, it resulted in an increase in their own emotional arousal, but only within those specific interventions and not across the sessions. It was found that clients did not immediately respond to challenges during interventions, but challenges tended to increase their emotional arousal over the course of the session. Furthermore, the study revealed an interaction effect between empathy and challenge, where sessions characterized by high or low levels of both empathy and challenge led to a decrease in heart rate and an increase in positive facial expressions. These findings underscore the therapeutic importance of empathy and challenge, providing support for the notion of a connected relationship between expressive interaction and individual emotion regulation within the therapeutic context.

Nødtvedt et al. employed hermeneutic-phenomenological thematic analysis to explore how clients perceive and experience the therapeutic relationship in emotion-focused therapy (EFT) for depression, anxiety, and severe self-criticism. Through interviews with 18 clients who underwent time-limited EFT, the researchers identified four main themes: the establishment of a trusting relationship or difficulties in connecting, collaboration and struggles in relating to painful emotions, managing alliance ruptures and the need for repair when working with distressing emotions, and acknowledging the significance of new relational experiences. The findings highlight the importance of therapists being genuine and establishing trust to enable clients to address vulnerability, and actively engage in EFT interventions. Moreover, therapists should consider individual client preferences and remain vigilant for potential alliance ruptures while working toward transforming negative emotions in therapy.

Ma et al. investigated the experiences of adolescents diagnosed with major depressive disorder who achieved positive treatment outcomes through acceptance and commitment therapy (ACT). Qualitative interviews revealed four main themes: the significance of the therapist's qualities and the therapeutic relationship, the importance of creating an environment for exploring and accepting negative emotions and practicing mindfulness, the value of engaging in actions aligned with personal values, and the impact of time settings on treatment. The findings emphasize the transformative effects that occur when adolescents have a therapist who is receptive and respectful, engage in self-exploration within a trusting therapeutic alliance, embrace acceptance of their thoughts and feelings, and live in the present moment. The study highlights the importance of targeting specific areas in the therapy process and guiding depressed adolescents toward effective recovery by focusing on behaviors that align with their core values.

Cheng et al. examined therapist-client linguistic mitigation in natural treatment settings by analyzing conversations between 15 clients and five therapists. The findings revealed that both therapists and clients employed various types of mitigation, with illocutionary mitigation and propositional mitigation being the most frequent. Therapists commonly used direct dissuasion, while clients used disclaimers at the most common subtypes of mitagators. The study also highlighted the cognitive-pragmatic functions of mitigation, including preserving positive face, maintaining social rights, and focusing on interactive goals. These functions contributed to building rapport in therapeutic conversations while reducing the risk of conflicts.

Yao and Zhang focused on how psychiatrists utilize conversational techniques, particularly by summarizing patients' perspectives on treatment, to address this challenge. Using Conversation Analysis, they examined face-to-face psychiatric consultations and found that patient formulation promotes mutual understanding and facilitates treatment decisions. It also has the potential to question the validity of patients' viewpoints, leading psychiatrists toward their preferred treatment direction. The authors argue that psychiatrists strive to achieve consensus by maintaining a balance between their institutional authority and consideration for patients' perspectives during the decision-making process.

This Research Topic provides readers with a comprehensive understanding of how psychotherapy is achieved through the interactions between therapists and clients. The research findings offer valuable insights into establishing a therapeutic alliance in real-life therapy sessions, spanning various types of therapy and shedding light on the factors that contribute to successful therapeutic outcomes.

## Author contributions

YW: Conceptualization, Methodology, Writing—original draft, Writing—review and editing.
